# Evaluation of the Duration of Good Visual Acuity During Anti-VEGF Therapy for Age-Related Macular Degeneration in Routine Clinical Practice

**DOI:** 10.3390/ijms262210927

**Published:** 2025-11-11

**Authors:** Andrea Gyenes, Lilla István, András Papp, Miklós Resch, Zsuzsa Récsán, Mónika Ecsedy, Zsuzsanna Szepessy, Antal Szabó, Balázs Lesch, György Barcsay, Ágnes Borbándy, Gábor László Sándor, Zoltán Z. Nagy, Illés Kovács

**Affiliations:** 1 Department of Ophthalmology, Semmelweis University, 1085 Budapest, Hungary; 2 Department of Ophthalmology, Weill Cornell Medical College, New York, NY 10021, USA

**Keywords:** nAMD, ‘time in range’, threshold, anti-VEGF, aflibercept, ranibizumab

## Abstract

The aim of this study was to analyse data from a clinical database using a novel visual acuity parameter to determine whether anti-VEGF molecules that target multiple domains involved in neovascularisation are more likely to achieve good visual acuity than agents that solely inhibit VEGF. This retrospective study analysed data from patients treated with anti-VEGF injections between 2015 and 2023. We set an ETDRS score threshold of 70 (equivalent to 20/40 Snellen acuity) to calculate ‘time in range’ (TIR). TIR is defined as ‘time spent with best-corrected visual acuity (BCVA) better than 20/40’ and can highlight significant variations in the time individuals spend above the threshold during their AMD treatment. Over nine years, 30,209 aflibercept and 10,876 ranibizumab injections were administered to 6043 patients. Patients received an average of 6.8 injections. The mean BCVA at the first injection was 57.00 ± 16.15 ETDRS letters for ranibizumab patients and 58.75 ± 15.82 for aflibercept patients, with a statistically significant difference (*p* < 0.001). Both groups showed significant improvement in visual acuity at follow-up (aflibercept: 60.21 ± 15.53; ranibizumab: 59.43 ± 15.81; both *p* < 0.001). The mean time between the two consecutive injections, including both the initial loading phase and the subsequent maintenance phase, was 67.22 ± 34.08 days for ranibizumab and 72.15 ± 31.00 days for aflibercept; the difference was statistically significant (*p* < 0.001). After controlling for the effect of initial BCVA and time between injections, patients who received aflibercept had a significantly higher average TIR (60.90 ± 36.27 days vs. 56.55 ± 38.78 days, *p* < 0.001), and significantly more likely to achieve >70 letters at the next visit (OR: 1.10; 95% CI: 1.05–1.15; *p* < 0.001) compared to patients receiving ranibizumab. Aflibercept treatment improves the likelihood of maintaining good BCVA by 10% compared to ranibizumab in patients receiving intravitreal anti-VEGF treatment for nAMD. Furthermore, the beneficial effects of aflibercept treatment are observed with less frequent dosing. Our results suggest that using anti-VEGF compounds that target multiple domains provides a detectable advantage in treating age-related macular degeneration, particularly when these agents have a longer duration of action.

## 1. Introduction

Age-related macular degeneration (AMD) is one of the leading global causes of vision loss in adults aged 50 years and older [1]. It affects approximately 30–50 million people worldwide, and visual impairment due to AMD significantly impacts health-related quality of life [2,3,4,5]. AMD affects quality of life in terms of tasks requiring good vision, such as reading and driving, as well as people’s ability to participate in social interactions and leisure activities [4,6,7]. The two main forms of the disease are neovascular and dry AMD [8]. VEGF plays a key role in neovascular AMD [9,10], and anti-VEGF agents, including bevacizumab, ranibizumab, aflibercept, brolucizumab and the dual-pathway anti-VEGF and angiopoietin-2 inhibitor faricimab, have been shown to inhibit the growth of neovascular lesions and resolve retinal oedema, resulting in positive visual outcomes [11,12,13,14,15,16]. In order to maintain the benefits of anti-VEGF therapy, most patients require regular treatment. However, the necessity for frequent injections and monitoring appointments represents a significant treatment burden, which often leads to undertreatment [17,18,19,20]. In recent years, the development of new drugs to treat age-related macular degeneration has focused on agents with multiple modes of action. The aim is to achieve stronger and more sustained therapeutic effects than those of traditional medications. Many clinical studies have examined treatment patterns and outcomes in patients with AMD; however, most of these studies have had limited follow-up periods ranging from one to two years [21,22,23,24]. Additionally, these studies have been affected by a high rate of patients lost to follow-up, raising concerns about the ability to generalize their findings to the wider population [24,25,26]. Furthermore, there is considerable diversity in clinical practice with regard to treatment patterns, which are based on a number of factors, including patients’ anatomical and visual responses to injections, clinician and patient preferences, and the range of available agents.

As AMD is a chronic disease with a lifelong prognosis, it is crucial to document patients’ long-term best-corrected visual acuity (BCVA) when they are undergoing intravitreal anti-VEGF injections. Furthermore, accurately assessing treatment response and disease control is essential for providing high-quality patient care and conducting clinical research [27,28]. In ophthalmology, most current functional endpoints describe the final state of a longitudinal clinical course, while fluctuations are often overlooked [29]. Although BCVA is used to demonstrate the efficacy of interventions, it is clear that it does not provide a full picture of overall visual performance. The novel concept of ‘time in range’ (TIR) has increasingly been used to assess glycaemic control in diabetic patients [30] and international normalised ratios in patients receiving anticoagulants [31]. This concept has recently been introduced to ophthalmology to measure the fluctuations in visual function associated with diabetic macular oedema [32]. As well as reflecting fluctuations in BCVA over time, TIR incorporates a BCVA threshold coinciding with a patient’s ability to perform certain tasks, such as driving and reading. Introducing and testing such a threshold enables us to better assess the impact of AMD treatment on visual function in terms of everyday activities. This study analysed data from a clinical database using this new visual acuity parameter to determine the effect of different intravitreal anti-VEGF injections for age-related macular degeneration in a clinical setting. Specifically, our study aimed to compare aflibercept—which targets multiple domains involved in neovascularisation, including VEGF-A, VEGF-B, and placental growth factor—with ranibizumab, which selectively inhibits VEGF-A, to determine whether these agents differ in their ability to achieve and maintain good visual acuity during treatment.

## 2. Results

**Figure 1:**
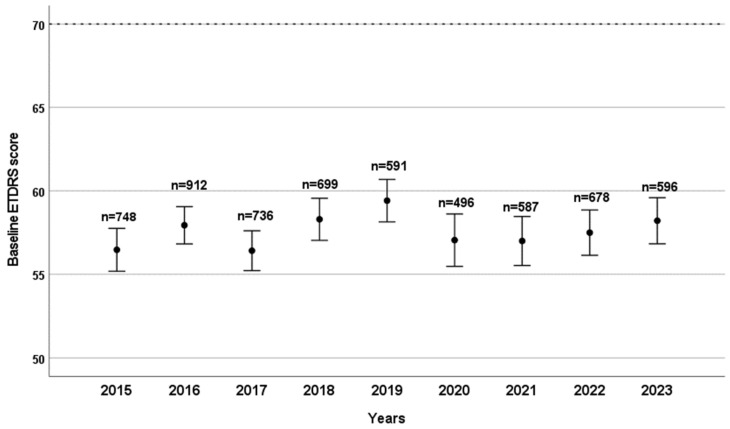
Baseline ETDRS visual acuity scores are shown by year, with the number of patients (n) who received their first intravitreal anti-VEGF treatment indicated for each year. Bars represent the 95% confidence interval of the mean score.

Over the course of nine years, a total of 30,209 intravitreal aflibercept and 10,876 intravitreal ranibizumab injections were administered at our outpatient clinic. The mean BCVA at the first injection was 57.00 ± 16.15 ETDRS letters for ranibizumab patients and 58.75 ± 15.82 for aflibercept patients; this difference was statistically significant (*p* < 0.001). Baseline BCVA prior to anti-VEGF injections did not show any significant differences over the years studied (Figure 1). The mean visual acuity of patients under treatment showed a significant improvement in both groups compared to the baseline average values (59.43 ± 15.81 ETDRS letter score for ranibizumab patients and 60.21 ± 15.53 for aflibercept patients; both *p* < 0.001).

**Figure 2:**
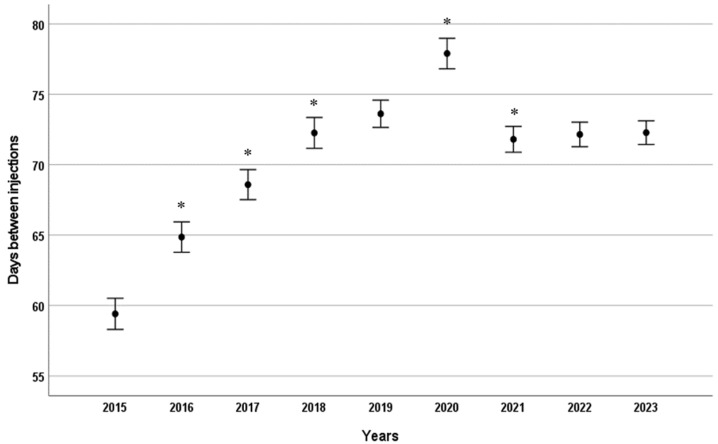
Mean time interval (days) between consecutive intravitreal anti-VEGF injections by year. Error bars represent 95% confidence intervals for the mean. * *p* < 0.001 compared to the previous year.

When analysing the data from the nine-year period, the mean time between the two consecutive injections, including the initial loading phase and subsequent maintenance phase, was found to be 67.22 ± 34.08 days for ranibizumab and 72.15 ± 31.00 days for aflibercept. There was a statistically significant difference between these two values (*p* < 0.001). Examining the data year-on-year, the interval between consecutive intravitreal injections (including initial loading and subsequent maintenance doses) increased steadily from 60 days to 72 days between 2015 and 2018, in line with evolving treatment protocols. This interval then remained stable, except for a spike in 2020, which was attributed to lockdown measures imposed due to the outbreak of the SARS-CoV-2 virus (Figure 2).

**Figure 3:**
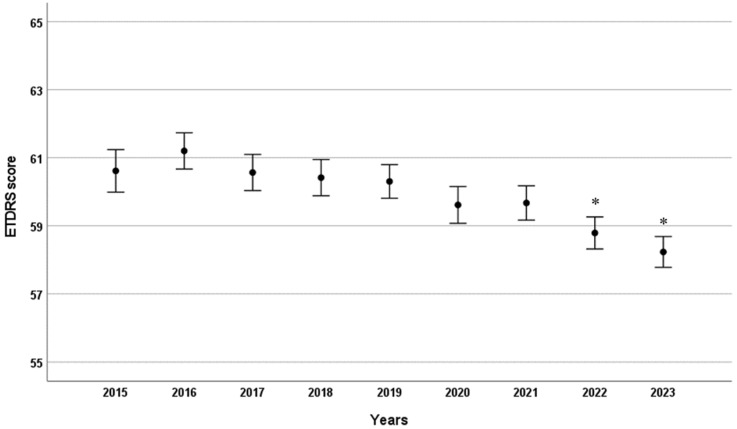
Mean ETDRS visual acuity scores by year in patients receiving intravitreal anti-VEGF injections. Error bars indicate the 95% confidence interval for the mean. * *p* < 0.001 compared to 2015.

There was no significant change in patients’ BCVA on the day of injection until 2021, despite the steady increase in time between consecutive injections (Figure 3). Additionally, there was no evidence that the less frequent dosing in 2020 impacted the BCVA of patients who received injections in 2020 or 2021 (Figure 3). However, a small but significant decrease in BCVA was observed in 2022 and 2023 compared to 2015–2019 (see Figure 3).

**Figure 4:**
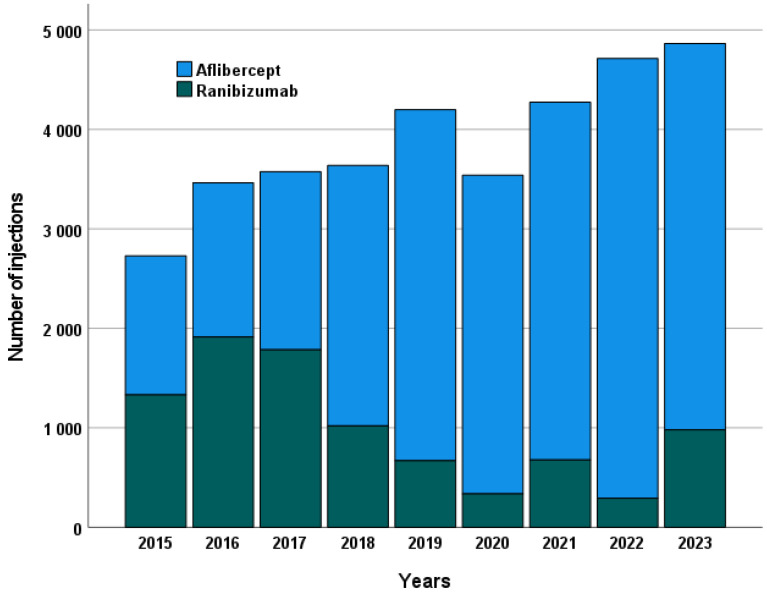
Annual number of intravitreal injections administered for each anti-VEGF agent, illustrating treatment frequency trends over the study period. The figure shows the total number of injections per year separately for ranibizumab and aflibercept, reflecting changes in treatment practice and adoption patterns of the two agents.

The total number of injections administered over the course of a year increased steadily throughout the study period. There was a continuous increase in aflibercept injections and a consistent decrease in ranibizumab injections (see Figure 4).

To calculate Time in Range (TIR), we identified all visits at which patients achieved a BCVA of at least 70 ETDRS letters on the day of examination. The time interval (in days) since the previous visit was then counted as the TIR value. For each patient, TIR was also expressed as a percentage, defined as the total number of days associated with visits showing BCVA ≥ 70 letters divided by the total days under observation. Patients in the aflibercept group had a significantly higher mean TIR (60.90 ± 36.27 days) compared to those treated with ranibizumab (56.55 ± 38.78 days, *p* < 0.001). When expressed as a percentage, TIR was 36.02% (217,759/604,589 days) for ranibizumab and 35.90% (672,706/1,873,916 days) for aflibercept, and this difference was not statistically significant (*p* = 0.09). Table 1 represents the proportion of patients with BCVA ≥ 70 expressed as a percentage annually, based on available visit-level data.

**Figure 5:**
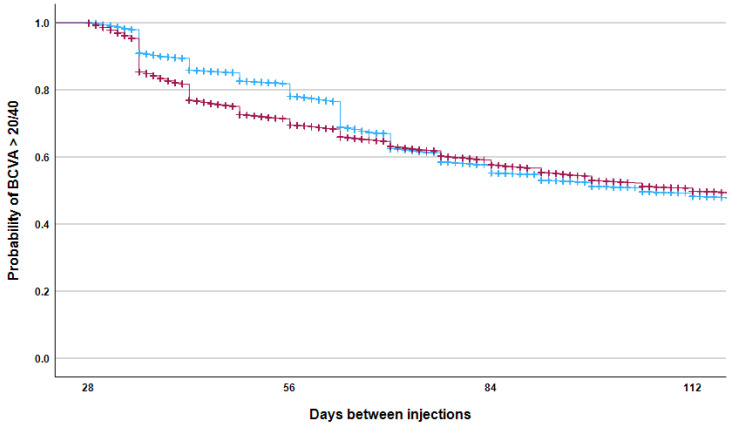
The Kaplan–Meier failure curve shows the probability of maintaining an ETDRS visual acuity score above 70 as a function of time between consecutive intravitreal injections with aflibercept (blue) and ranibizumab (red). The difference between the groups was statistically significant (log-rank test, *p* < 0.001).

**Figure 6:**
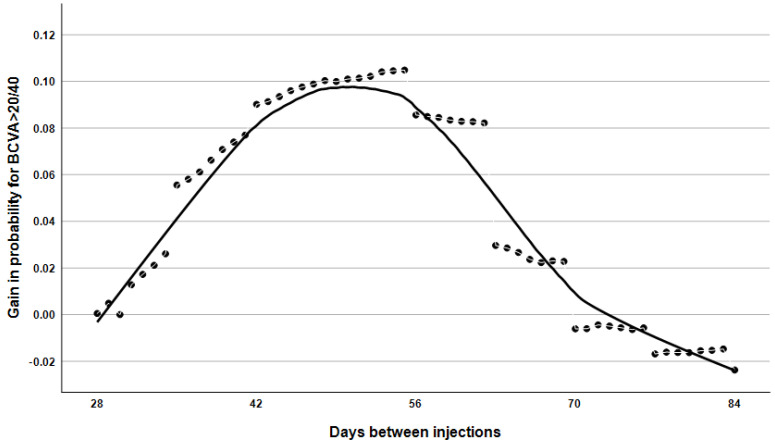
The probability curve depicts changes in the likelihood of achieving an ETDRS score greater than 70 between consecutive injections with aflibercept compared to ranibizumab.

However, Kaplan–Meier analysis (Figure 5) showed that aflibercept treatment was associated with a greater probability of maintaining BCVA ≥ 70 ETDRS letters, especially when the interval between injections was between 35 and 70 days. When analyzing data from patients with ≤120 days between consecutive visits, those receiving aflibercept remained significantly more likely to achieve BCVA ≥ 70 ETDRS letters at the next visit, even after adjusting for baseline BCVA and the inter-visit interval (OR: 1.10, 95% CI: 1.05–1.15, *p* < 0.001). In the clinically relevant 4–12-week window, aflibercept demonstrated a clear advantage in maintaining BCVA ≥ 70 ETDRS letters at the 6–8-week treatment interval (Figure 6).

Figure 6 demonstrates that aflibercept treatment was superior to ranibizumab in enabling patients to achieve a minimum visual acuity of 70 ETDRS letters within the 28–84 day post-treatment period. This advantage is most pronounced at the 6–8 week injection interval and gradually diminishes as the interval between treatments increases.

### Safety

Finally, we examined the safety of intravitreal anti-VEGF treatment in relation to post-interventional endophthalmitis in our sample. Our results showed an overall prevalence of endophthalmitis of 0.049% (20 cases out of 41,225), ranging from 0.018% to 0.14% in annual breakdowns. No difference in the prevalence of endophthalmitis was found when comparing the two types of drug (*p* > 0.05).

## 3. Discussion

In this study, we analysed data from a large database to determine time-in-range, which is defined as ‘time spent with BCVA better than 20/40’, in patients receiving intravitreal anti-VEGF injections for age-related macular degeneration in a clinical setting. Initially, no statistically significant differences were observed in the efficacy of the two active substances when the proportion of the period with good visual acuity relative to the total treatment period was examined. This examination was conducted for treatment intervals ranging from 4 to 16 weeks, and patients demonstrated a visual acuity of at least 70 ETDRS letters 36% of the time. This result is important because, even if a patient only has one good eye, they can still be expected to be able to drive and read for this proportion of time during treatment. As many patients are of working age, this is crucial for everyday social and work activities. Patients whose initial visual acuity was above this threshold were, of course, more likely to remain within this range throughout their treatment than those whose initial visual acuity was poor. Furthermore, the beneficial effects of aflibercept treatment are also observed with less frequent dosing, as our data revealed a significant difference in the frequency of injections. However, upon examination of the relationship between treatment interval and good visual acuity, it was found that aflibercept treatment, within the 28- to 84-day interval (which corresponds to the conventional dosing regimen in clinical practice), was associated with a 10% higher probability of achieving a minimum ETDRS visual acuity of 70 letters. When the follow-up interval is less than 28 days, it is not possible to meaningfully compare the effects of the two injections, given the fixed minimum follow-up period of four weeks. Nevertheless, no major differences were observed in the impact of the two intravitreal injections when administered more than 84 days apart. This is likely because the disease was in a phase of inactivity or scarring at the time. Although the difference in efficacy between the two drugs does not appear substantial, the analysis of such a large dataset enables the detection of trends that would not be observable in smaller samples, thereby identifying potential targets for future studies. In our cohort, despite patients receiving treatment less frequently during the lockdowns imposed due to the pandemic, we note that their BCVA did not decrease during the same period. However, we observed a slight decrease in BCVA in 2022 and 2023 compared to previous years, which can be explained by the delayed effects of less frequent injections or vision loss due to cataract surgeries being postponed because of lockdowns.

As the database we used did not contain retinal thickness data, we were unable to study the relationship between morphological and functional changes. Although contrast sensitivity and microperimetry testing also show significant correlations with activities of daily living task tests performance in AMD patients, monocular BCVA remains the most common measure of visual function [33,34]. The most prevalent functional endpoint in ophthalmic clinical trials is the alteration in BCVA, with secondary functional endpoints being 5-, 10-, and 15-letter gainers and losers, as well as meaningful VA levels such as 20/40 and 20/200 [35]. The BCVA threshold of 20/40 is the most universally used visual threshold test for obtaining a driver’s licence, yet the BCVA requirements vary from country to country [36].

In our dataset, patients who received repeated injections of either ranibizumab or aflibercept experienced an average improvement in ETDRS score of approximately 1.5 letters compared to their baseline BCVA. Although there was a significant difference in vision improvement between the two drugs, we do not consider a difference of only one letter to be clinically significant; this difference was only statistically significant due to the large amount of data analysed. While this is less than the improvement in BCVA reported in randomised clinical trials (RCTs), our results are based on data from a large-scale clinical setting. In our analysis, the mean change in ETDRS score across all patients does not fully reflect the duration for which patients can maintain functional vision, defined as ≥70 ETDRS letters. The distinction between these metrics is critical; whereas mean BCVA quantifies overall cohort improvement, sustained achievement of the 70-letter threshold better captures clinically relevant outcomes. Therefore, it is important to note that an increase in mean visual acuity does not necessarily translate into prolonged maintenance of good functional vision among patients. It is not uncommon for therapeutic agents to demonstrate high efficacy in phase III clinical trials but lower efficacy in real-world clinical practice. According to the findings of the integrated VIEW 1 and VIEW 2 studies, patients treated with aflibercept experienced an average improvement in BCVA of 8.4 letters after 52 weeks of treatment [15]. The real-world data, however, suggests a less marked improvement [37,38,39,40,41]. The pivotal ranibizumab trials MARINA [12,42,43] and ANCHOR [13,44] showed that patients who received the treatment once a month gained an average of 7 to 11 letters over 12 months. Researchers studied how ranibizumab worked in real-world conditions in several countries. They found that, just like with aflibercept, the BCVA results were much worse than those reported in the important MARINA and ANCHOR trials [45].

In contrast to the results from a few hundred patients who were selected for the RCTs based on strict inclusion criteria and who received frequent treatment, usually for up to two years, in our study, we had BCVA data from a large number of patients who started, stopped, or discontinued treatment over a period of nine years. In the present cohort, the retinal specialist elected to discontinue treatment in accordance with the relevant protocols when no further benefit was anticipated. This includes cases that improved with treatment, or stagnated or worsened despite treatment, which cannot be identified and analysed separately in this dataset. It is hypothesised that the visual function of the patients in the cohort may have improved by more than the calculated 1.5 ETDRS letters during the first year or two; however, this improvement gradually decreased in the following years, when the patients continued to receive treatment to preserve their vision despite the deterioration of their visual function. Evidence from clinical practice indicates a decline in the efficacy of anti-VEGF treatment and, consequently, a decrease in BCVA among patients over time. Moreover, the VA enhancement demonstrated in the RCT does not persist in the long term. In contrast to randomized controlled trials, where visual outcomes are typically evaluated as changes between consecutive visits, our approach provides a broader, population-level perspective. The analysis is based on aggregated real-world data rather than structured treatment protocols, allowing a more comprehensive assessment of long-term treatment effectiveness across a large and heterogeneous cohort. Although this method lacks the granularity of visit-by-visit analyses, it offers valuable insight into how anti-VEGF therapy maintains functional vision over extended periods under everyday clinical conditions.

In general, larger studies provide stronger and more reliable results because they have smaller margins of error and lower standard deviations [46,47]. Our results are in line with previous results from large patient registries. In a 2021 study of IRIS (Intelligent Research in Sight) Registry data, MacCumber et al. observed that patients with nAMD who underwent up to 3.5 years of follow-up treatment received, on average, 5.6 injections in the first year, 3.4 in the second year, and 3.1 in the third year [48]. The IRIS Registry database is the first US-based national comprehensive eye disease database and is the largest ophthalmic registry worldwide based on electronic health records. The IRIS Registry is a prominent data source for patients in the US, as it collates information from approximately 70% of ophthalmology practices across all payers and all populations, including those without insurance [49]. An analysis of data from the IRIS Registry revealed a marginal improvement in BCVA of 0.7 ETDRS letters from baseline at the end of year one (baseline mean: 55.4 letters). However, by the end of year 3, the mean BCVA had decreased by 3.1 letters from the baseline. Furthermore, it was observed that more than one-third of subjects had discontinued treatment by the end of year 3. These results are consistent with the findings of the current study. It is important to acknowledge that the IRIS registry study was predominantly descriptive in nature. It did not examine the occurrence of treatment gaps or discontinuation, the underlying causes of sustained poor vision or considerable vision loss, or the potential association between the frequency of intravitreal anti-VEGF injections, patient baseline characteristics, and BCVA outcomes in neovascular age-related macular degeneration. Recently, Wyckoff and colleagues examined the same database [50] and found that following an initial increase of 3.0 ETDRS letters at one year, there was a net loss of 4.6 letters from the baseline at six years. Based on a cohort of 254,655 eyes (226,767 patients) with up to 6 years of follow-up, they found that from a mean of 7.2 in year 1 and 5.6 in year 2, the mean number of injections plateaued between 4.2 and 4.6 in years 3 through 6. In their database, 38.8% of eyes underwent treatment cessation, while 32.3% underwent a change in treatment. Of the patients, 58.5% lost at least 10 letters of BCVA at least once during follow-up, with 14.5% of patients experiencing sustained poor vision after a median of 3.4 years.

When evaluating our results, it should be noted that we hypothesise, in accordance with clinical experience, that some patients may have received regular intravitreal injections for several years until the exudative process became inactive. The findings of this study underscore the significance of optimal management during the maintenance phase of anti-VEGF treatment algorithms. Rigorous control of retinal volume fluctuations is imperative to avert the decline in visual acuity over time. The utilisation of medications characterised by prolonged duration of action and sustained release mechanisms is hypothesised to result in superior visual outcomes, concomitant with a reduction in treatment burden [51].

### 3.1. Pharmacodynamic Characteristics of Different Therapeutic Agents

Due to physiological barriers, achieving therapeutic drug levels in the vitreous body via topical or systemic administration is challenging; thus, intravitreal injection remains the most effective route for posterior segment diseases [52]. Anti-VEGF agents differ significantly in their molecular properties, including size and structure, which directly impact their pharmacokinetics—particularly their distribution and clearance from the vitreous body. Understanding these half-lives is critical for optimizing treatment intervals and maximizing therapeutic efficacy while minimizing treatment burden. In clinical practice, anti-VEGF dosing regimens are not standardized. Fixed regimens, involving monthly or bimonthly injections, have shown superior visual outcomes in clinical trials but are rarely implemented due to logistical and economic burdens [12,15,44,53,54]. Two commonly used regimens are pro re nata (PRN), based on disease reactivation, and Treat-and-Extend (T&E), a proactive approach aimed at minimizing injection frequency [55,56]. T&E begins with three monthly loading doses, followed by interval extension if no disease activity is observed; intervals are shortened upon recurrence. T&E achieves comparable visual outcomes to monthly dosing with fewer injections, and improved visual gains over PRN, thus with slightly more injections [57]. Ideally, dosing frequency should align with the half-life of the drug in order to achieve a sustained therapeutic drug concentration in the vitreous body. However, direct measurement of intraocular concentrations requires invasive methods, limiting such studies to preclinical settings [58,59]. Consequently, most human studies calculate a serum half-life [60,61] or even an aqueous half-life, which could be an indirect estimate of the half-life of the antibodies in the vitreous humour [62]. The vitreous half-life on rabbits (the most studied animal) is, on average, 4.94 days for bevacizumab, 2.83 days for ranibizumab and 4.58 days for aflibercept. Although the relation between the antibody intravitreal half-life and the molecular weight is not always clear, in this case, it follows the general rule that the intravitreal half-life of macromolecules has a direct correlation with the molecular weight (MW). In this way, bevacizumab (MW = 150 kDa) presents the highest vitreous half-life of the three antibodies, followed by aflibercept (MW = 115 kDa), which has a slightly lower intravitreal half-life, and ranibizumab (MW = 48 kDa) with the lowest one [63]. The unique anatomical and physicochemical characteristics of the vitreous body impact the diffusion, bioavailability, and clearance of therapeutic agents, particularly large molecules such as anti-VEGF drugs. The vitreous gel has a strong net negative charge, attributed to components like hyaluronic acid. The negatively charged vitreous matrix can modulate the movement of anti-VEGF drugs via electrostatic interactions. These interactions depend on the physicochemical properties of each drug, particularly their net charge. For example, ranibizumab is negatively charged at physiological pH, allowing unimpeded diffusion in the vitreous body. Aflibercept’s mild positive charge may cause increased interaction with the vitreous matrix, affecting its pharmacokinetics [62]. Moreover, with aging, the vitreous body undergoes liquefaction, a factor that may influence molecular diffusion and clearance [62].

The molecular size and charge of anti-VEGF agents have important influences on distribution in and elimination from the vitreous body and on peak drug efficacy [62,64]. Drug elimination from the vitreous body occurs either via metabolism or systemic clearance; however, anti-VEGF agents undergo minimal intraocular metabolism or degradation [65,66]. Following intravitreal injection, clearance proceeds through two main routes. The anterior route, via diffusion into the aqueous humour and subsequent outflow, is considered the primary pathway for anti-VEGF elimination [60,67,68,69,70]. The posterior route, involving secretion through the ciliary body, iris, or retinal pigment epithelium, remains less defined [66].

There are strategies to increase the drug duration of action, including modification of charge, valence, neonatal fragment crystallizable (Fc) receptor (FcRn)-binding affinity, and glycosylation [71]. Fc fragment (fragment crystallizable) of the antibody can bind to Fc receptors on cells to illicit immune effector functions or to the neonatal Fc receptor (FcRn) to protect it from degradation [72]. Furthermore, binding of antibodies to FcRn on epithelial and endothelial cells through their Fc domain regulates their metabolism and partially accounts for their relatively long serum half-lives [73,74].

Ranibizumab is a 48-kD Fab fragment of the A4.6.1 antibody, which is one of the four antibodies of the IgG1 isotope that most effectively binds and neutralizes all VEGF-A isoforms (VEGF121, VEGF165, VEGF189). It is approved for neovascular AMD based on the ANCHOR and MARINA trials [12,44]. Ranibizumab lacks the Fc region, allowing it to avoid Fc recycling and making it significantly smaller than the full-size antibody. The smaller size is thought to facilitate easier penetration into the retina and faster clearance systemically; however, this may also expedite clearance from the vitreous body.

Aflibercept is a fully human recombinant fusion protein that inhibits VEGF-A, VEGF-B, and placental growth factor (PlGF) [75,76,77]. Reduced VEGF activity leads to decreased angiogenesis and vascular permeability; inhibition of PlGF and VEGF-B may also aid the treatment of angiogenic conditions. It consists of an IgG backbone fused to extracellular VEGF receptor sequences of the human VEGFR1 and VEGFR2. Aflibercept has a unique binding action and binds to both sides of the VEGF dimer, forming an inert 1:1 complex, also termed a VEGF trap. Initially administered as 2 mg, the efficacy of aflibercept in treating nAMD, particularly in improving BCVA and reducing central retinal thickness. Multiple studies have shown it to be either non-inferior or potentially superior to other anti-VEGF agents, as well as allowing for an extended dosing interval while maintaining a similar safety profile [78,79,80,81]. Aflibercept, licensed in the USA in 2011 and in Europe in 2012, for the treatment of wet-type AMD. The success of aflibercept has led to the exploration of a higher dosage. According to preliminary data, an 8 mg dose could extend the duration of VEGF inhibition [82,83]. CANDELA and PULSAR trials have focused on the safety and anatomical efficacy of the 8 mg dose in nAMD [84,85,86]. These results contributed to the approval of the 8 mg dose by the U.S. Food and Drug Administration (FDA) in August of 2023.

The newer agent faricimab is a bispecific antibody built on a human IgG1 framework developed to simultaneously inhibit VEGF-A and Ang-2, with the goal of promoting vascular stability in retinal vascular diseases such as AMD and diabetic retinopathy [87,88]. The Ang/Tie pathway regulates vascular stability, angiogenesis, permeability, and inflammation [89,90]. Deletion of the Fc gamma receptor and neonatal Fc receptor binding site prevents intracellular immunoglobulin G recycling, thereby reducing the systemic half-life of faricimab and reducing systemic exposure [87]. The phase 3 TENAYA and LUCERNE trials aimed to assess efficacy, safety, and durability of faricimab, the first bispecific antibody for intraocular use [91]. TRUCKEE study highlights the benefit of faricimab on real-world patients with nAMD [92]. Of all the anti-VEGF drugs currently in use, faricimab offers the longest treatment interval.

### 3.2. Limitations

Despite the considerable potential for meaningful analysis offered by registry data, there are inherent challenges in drawing appropriate conclusions. It should be noted that data on anatomical outcomes, such as macular fluid status as determined by OCT, were not available for inclusion in this analysis. It is important to note that fluctuations in central retinal subfield thickness (CSFT) are likely to have an impact on visual acuity outcomes. Nevertheless, comparative effectiveness trials evaluated average outcomes and did not take the severity of the fluctuation into consideration. Furthermore, they did not test the trajectory of vision loss by the severity of fluctuation. The standard deviation (SD) of an OCT metric, measured at multiple visits, can be regarded as an indicator of repeated cycles of retinal thinning and thickening. This is due to the fact that the SD can be considered as a marker for the severity of thickness fluctuations over time [51]. There are further indicators of a biological or pathogenic process that has an indirect influence on visual function. It is important to note that structural endpoints do not directly measure a clinically meaningful benefit [34,93].

Due to the nature of the available data in this study, it was not possible to determine the extent of visual improvement based on the number of injections or treatment duration. In our cohort, treatment regimens changed over the nine-year study period, evolving from an initial fixed dose to PRN and then to a T&E protocol. It should be noted that, at the time of the registry analysis, not all patients had necessarily received all injections in accordance with the current protocol. In such cases, the data are said to be censored, meaning that the registry observation period ended before all events (e.g., injections) occurred. In such cases, it is uncertain whether the next injection will occur at all. In addition, a registry may enrol patients until a predetermined stopping date. Consequently, patients enrolled earlier in the registry are more likely to have received more injections than those enrolled more recently due to the longer follow-up period.

The analysis was limited by the lack of information on the number of patients who may have missed visits. The reasons for treatment discontinuation or gaps in treatment, such as poor or no response to treatment, were not available. The natural progression of neovascular AMD is typically unfavorable [94]; consequently, it is reasonable to expect a decline in visual acuity among patients who discontinued treatment over time. Another limitation is that the timing and reasons for any medication changes that may have occurred cannot be identified. Due to considerable variability in injection treatment patterns (including number of anti-VEGF injections, treatment intervals and switching of agents) from year to year, these patterns could not be reliably related to outcomes at the end of the follow-up period. Furthermore, conditions other than AMD (e.g., glaucoma and cataracts) could have led to poor visual acuity and may not have been captured in the current study. However, we can reasonably assume that, in such a large database, their occurrence over the years and in relation to the two active substances under review shows a similar distribution. Therefore, statistical comparisons between groups and over time provide a realistic estimate of the population.

The methodology used to calculate time in range is also limited, particularly when applied to progressive diseases and continuous endpoints. While all available data were used for the calculation, BCVA was not assessed daily and it was assumed that patients had maintained the same BCVA value prior to the current measurement. Therefore, the data and their interpretation should be treated with caution, as they may not be entirely accurate. However, clinical practice shows that the visual benefit of anti-VEGF injections is most pronounced in the first weeks after administration, after which vision gradually deteriorates due to the cessation of the drug’s pharmacological effects. Based on this knowledge, it is reasonable to assume that patients with BCVA better than 70 ETDRS letters on the day of injection may have exceeded this limit in the period following the previous injection.

As the dataset does not indicate at which injection number each patient was at a given visit, we were unable to perform a longitudinal analysis and instead focused on cross-sectional comparisons. This approach was deliberate, as our objective was not to follow individual patient trajectories over time but to provide an overall, population-level snapshot—an analysis complementary to longitudinal studies and randomized controlled trials. The yearly means thus reflect the aggregate experience of all patients treated each year, rather than repeated measures from the same cohort.

It is acknowledged that the aforementioned limitations may have exerted an effect on the results obtained from the treatment of all patients to an equivalent degree. Consequently, it is hypothesised that the conclusions drawn from the database analysis are accurate in terms of the comparison of the efficacy of the two drugs. Despite these weaknesses, our registry can provide insight into the effectiveness and safety of anti-VEGF injections, as well as the efficiency, timeliness, quality and patient-centredness of a healthcare system.

## 4. Materials and Methods

This retrospective study analysed data from patients treated with anti-VEGF therapy for age-related macular degeneration at the Department of Ophthalmology at Semmelweis University between 1 January 2015 and 31 December 2023. Data were obtained from the Hungarian National Health Insurance Fund database. Table 2 summarizes the inclusion and exclusion criteria applied for patient selection in this study. The criteria are based on the approved Summary of Product Characteristics (SPCs) for intravitreal aflibercept and ranibizumab in the treatment of neovascular (wet) age-related macular degeneration (AMD) according to national and European guidelines.

The data available for retrospective analysis covered a period of nine years. This included the following information: the date of treatment; ETDRS visual acuity measured on the day of treatment; the type of anti-VEGF substance used; the time (in days) since the previous treatment; and whether the treatment was the first or a repeat. BCVA was measured at each visit using the standardized ETDRS chart protocol at 4 m, with results recorded as the total number of letters correctly identified by the patient. However, the dataset did not include information on the number of previous injections received by the patient, their BCVA prior to the current treatment, or their age, sex, and other medical conditions (Table 3).

### Statistical Calculations

Since the observations within this dataset could not be considered fully independent, an appropriate statistical approach was applied to ensure the reliability of *p*-values. The Bonferroni correction—the most commonly used procedure in this context—was used to adjust significance thresholds and control the risk of type I error due to multiple comparisons. The mean number of injections per patient was determined from the ratio of initial and repeat injections. Statistical significance was first evaluated at a conventional *p*-value threshold of 0.05, then adjusted using the Bonferroni method for all subsequent analyses. Multiple linear regression was performed to identify factors associated with visual acuity (VA) measured on the day of injection. Although mixed-effects models are generally preferable for repeated measures because they account for both fixed and random effects, our dataset did not reliably link repeated observations to individual patients, precluding the correct specification of the correlation structure required for such models. Therefore, we used multiple linear regression with Bonferroni correction to control for type I error, as this approach provided interpretable results and better suited the data structure and available covariates. For Time in Range (TIR) calculations, the ETDRS letter score threshold was set at 70, which corresponds approximately to a Snellen BCVA of 20/40—the legal limit for driving in many countries. TIR was calculated as the time (in days) between qualifying visits where patients achieved BCVA ≥ 70 letters. If a patient achieved a visual acuity of ≥70 letters at a given visit, the time interval (in days) since the previous visit was considered as the TIR value. Thus, TIR reflects the total time during which vision remained better than 20/40, highlighting differences in visual outcomes throughout AMD treatment. Statistical analyses were conducted using Statistica 14.3 (StatSoft Inc., Tulsa, OK, USA), and all data are presented as mean ± standard deviation or with the associated 95% confidence intervals.

## 5. Conclusions

One of our key findings is that, for any day between days 28 and 84, patients receiving aflibercept have a significantly higher probability of achieving a BCVA of over 70 letters than those receiving ranibizumab. The benefits of aflibercept were most noticeable when administered every 8 weeks; thereafter, they gradually decreased as the treatment interval increased. This observation lends weight to the development of new, prolonged-action anti-VEGF agents (e.g., aflibercept HD and faricimab). Our findings indicate that anti-VEGF agents designed to target multiple binding sites exhibit superior therapeutic efficacy compared with single-site inhibitors. By engaging several domains of the VEGF pathway, these agents achieve a more comprehensive suppression of pathological angiogenesis and vascular hyperpermeability. From a molecular biology perspective, multi-site inhibition represents a particularly promising strategy for the durable attenuation of abnormally elevated intraocular VEGF levels. This prolonged blockade has the potential to reduce dosing frequency, limit treatment burden, and ultimately improve long-term anatomical and functional outcomes in VEGF-driven ocular diseases. Based on randomised clinical trials conducted for registration purposes, the treatment interval for these latter agents can be extended significantly to 3–5 months following the loading dose. In the future, it will be worth investigating whether good visual acuity can be maintained at the same rate with this frequency of administration. Despite the limitations of the data, health registries such as those used in this study can provide insight into the effectiveness and safety of anti-VEGF injections, as well as the efficiency, timeliness, quality, and patient-centredness of healthcare systems.

## Figures and Tables

**Table 1:** Proportion of patients with BCVA ≥ 70 per drug annually.

Drug	2015	2016	2017	2018	2019	2020	2021	2022	2023
Ranibizumab	35.2%	36.6%	32.9%	35.7%	29.0%	31.1%	32.7%	31.3%	29.9%
Aflibercept	37.2%	37.6%	36.8%	38.6%	38.2%	35.8%	36.5%	35.1%	33.2%

**Table 2:** Inclusion and exclusion criteria applied for patient selection and treatment in the current AMD cohort.

Criterion Type	Description
Inclusion Criteria	Age ≥ 50 years
	Clinical diagnosis of active neovascular AMD (confirmed by imaging)
	Eligible for intravitreal anti-VEGF therapy per national/European guidelines
	Adequate documentation and follow-up in national registry
Exclusion Criteria	Ocular or periocular infection (active or suspected)
	Known hypersensitivity (allergy) to anti-VEGF agents or excipients
	Severe intraocular inflammation
	Recent (<28 days) or planned intraocular surgery
	Retinal tear or detachment not fully repaired
	Subretinal hemorrhage affecting the fovea or ≥50% of lesion area
	Marked decrease in visual acuity (≥30 ETDRS letters from prior visit)
	Intraocular pressure ≥ 30 mmHg (especially for ranibizumab)
	Prior anti-VEGF for other indications (e.g., DME, RVO)
	Incomplete documentation or missing outcome/diagnostic data

**Table 3:** Available and unavailable data parameters in the study database.

Data Element	Available in Database	Not Available in Database
Date of treatment	✓	
ETDRS BCVA measured on treatment day	✓	
Type of anti-VEGF agent used	✓	
Time (days) since previous treatment	✓	
First or repeat treatment	✓	
Number of previous injections		✓
BCVA prior to current treatment		✓
Patient age		✓
Patient sex		✓
Other medical conditions		✓

## Data Availability

Anonymized data supporting this study are available from the corresponding author upon reasonable request, subject to ethics committee approval and compliance with institutional data sharing policies.

## Abbreviations

The following abbreviations are used in this manuscript:

nAMD	neovascular Age-related Macular Degeneration
VA	Visual Acuity
BCVA	Best-corrected Visual Acuity
VEGF	Vascular Endothelial Growth Factor
PlGF	Placental Growth Factor
T&E	Treat-and-Extend
PRN	Pro re nata
TIR	Time in range
OR	Odds ratio
CI	Confidence interval
